# Targeting RNA splicing modulation: new perspectives for anticancer strategy?

**DOI:** 10.1186/s13046-025-03279-w

**Published:** 2025-01-30

**Authors:** Xuemei Lv, Xiaoyu Sun, Yang Gao, Xinyue Song, Xiaoyun Hu, Lang Gong, Li Han, Miao He, Minjie Wei

**Affiliations:** 1 Department of Pharmacology, School of Pharmacy, China Medical University, No.77 Puhe Road, Shenyang North New Area, Shenyang, Liaoning Province 110122 P. R. China; 2 Central Laboratory, School of Pharmacy, China Medical University, Shenyang, Liaoning Province China; 3 Liaoning Key Laboratory of Molecular Targeted Anti-Tumor Drug Development and Evaluation, Liaoning Cancer Immune Peptide Drug Engineering Technology Research Center, Shenyang, China; 4 Shenyang Kangwei Medical Laboratory Analysis Co. LTD, Shenyang, China; 5 Scientific Experimental Center, School of Pharmacy, China Medical University, Shenyang, 110122 P. R. China

**Keywords:** Cancer, Alternative splicing, Splicing modulators, Splice-switching ASO, Targeted therapy

## Abstract

The excision of introns from pre-mRNA is a crucial process in the expression of the majority of genes. Alternative splicing allows a single gene to generate diverse mRNA and protein products. Aberrant RNA splicing is recognized as a molecular characteristic present in almost all types of tumors. Therefore, identifying cancer-specific subtypes from aberrant processing offers new opportunities for therapeutic development. Numerous splicing modulators, each utilizing different mechanisms, have been developed as promising anticancer therapies, some of which are in clinical trials. In this review, we summarize the splice-altered signatures of cancer cell transcriptomes and the contributions of splicing aberrations to tumorigenesis and progression. Especially, we discuss current and emerging RNA splicing-targeted strategies for cancer therapy, including pharmacological approaches and splice-switching antisense oligonucleotides (ASOs). Finally, we address the challenges and opportunities in translating these findings into clinical practice.

## Introduction

RNA splicing is an essential process in the expression of most human genes, which involves removing introns from precursor messenger RNA (pre-mRNA) and joining exons to produce mature mRNA [1]. Most multi-exonic human genes undergo alternative splicing (AS), generating distinct mature mRNAs from a single primary transcript and expanding the protein-coding repertoire [2, 3]. Most tumors exhibit extensive splicing abnormalities, including abnormal retention of introns that are usually excised, inappropriate expression of isoforms, and either inactivation of tumor suppressors or promotion of oncogene expression [4–6]. Therefore, identifying cancer-specific subtypes from aberrant splicing offers new opportunities for developing cancer therapeutics. Currently, small molecule inhibitors targeting oncogenic splicing factors or splicing machinery elements are being developed for anticancer therapy [7]. Additionally, individualized splice-switching antisense oligonucleotide (ASO) therapy provides a novel and personalized approach to cancer treatment [8].

This review focuses on the basic mechanisms of RNA splicing and its dysregulation in cancer. And it discusses current and emerging strategies to target RNA splicing, including the pharmacological regulation of RNA splicing and advances in ASO-targeted RNA splicing in cancer therapy. First, the molecular mechanisms of pre-mRNA splicing are introduced, followed by a discussion on the complex interactions between regulators that determine the splicing sites of pre-mRNA. Next, we describe how the dysregulation of splicing in cancer leads to the expression of aberrant mRNA isoforms associated with “cancer hallmarks”, which promote tumor progression. Finally, the review discusses the future of pre-mRNA processing research in anticancer drug discovery, emphasizing the potential of splicing-derived neoantigens to enhance immunotherapy and the development of drugs that target these splicing.

## Basic mechanisms of RNA splicing and regulation

**Fig. 1:**
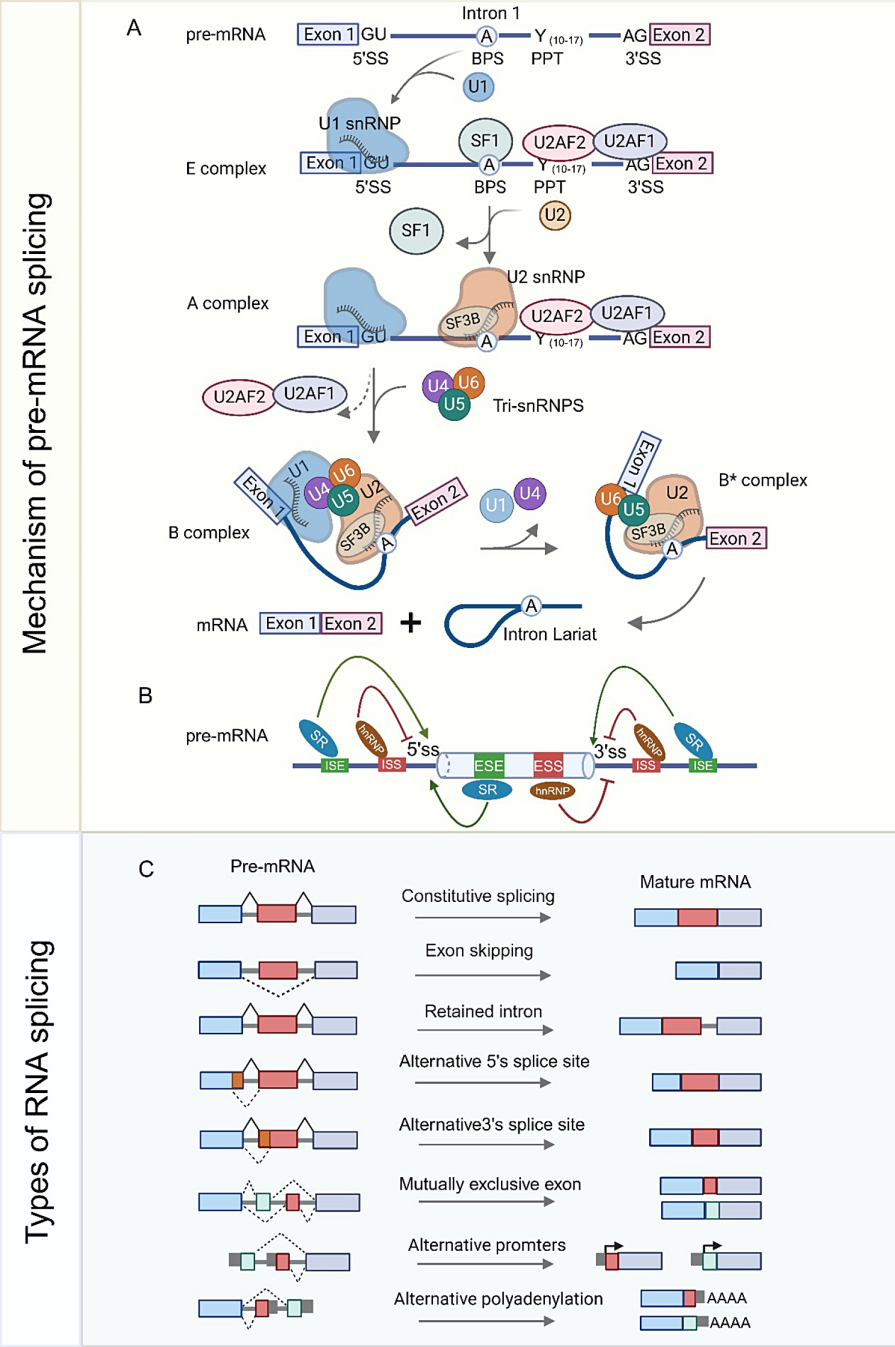
The regulation mechanism and mode of RNA splicing. (**A**) Pre-mRNA includes 5’ splice sites (5’ ss), 3’ splice sites (3’ ss), branch point sites (BPS), and (BPS), and polypyrimidine channels (PPT). U1 snRNP recognized 5’ss, and SF1 bound BPS, U2AF2 and U2AF1, PPT, and 3’ss to form the E complex, respectively. U2 snRNP replaces SF1 and binds to BPS to form an A complex, which subsequently recruits U4, U5, and U6 triple snRNP complexes. The B complex is rearranged to form catalytically activated complex B, which is followed by two transesterification reactions catalyzing the eventual formation of mature mRNA and the intron Lariat. (**B**) Cis-regulatory elements in pre-mRNA interact with splicing factors to regulate the splicing process. SR proteins act as splicing activators and promote splicing by binding to ESEs and ISEs. HnRNPs act as repressors and inhibit binding to splice sites by interacting with ESSs and ISSs. (**C**) RNA splicing is composed of constitutive splicing and AS. AS including exon skipping, retention intron, alternative 5’ splicing, alternative 3’ splicing, mutually exclusive exon, alternative promoters, and alternative polyadenylation. Exons are represented by boxes, and introns by lines. Promoters are indicated by arrows, and polyadenylation sites are indicated by AAAA. This figure was drawn by Biorender

RNA splicing is regulated by the spliceosome, a substantial ribonucleoprotein complex composed of five small nuclear RNAs (snRNAs: U1, U2, U4, U5, and U6) and approximately 200 associated proteins [9]. In the process of splicing, all sorts of spliceosomes (including the E complex, A complex, B complex, B* complex, etc.) play an important role (Fig. 1A). The spliceosome identifies essential regulatory sequences in pre-mRNA, such as the 5′ splice donor site (5′ss) and 3′ splice acceptor site (3′ss), which define intron-exon boundaries. It also recognizes the branch point site (BPS) and the polypyrimidine tract (PPT) located between the BPS and the 3′ss [10]. The U1 snRNA molecule inside the U1 snRNP complex forms a base complementary pairing with the GU dinucleotide located at the 5’ end of the pre-mRNA. Splicing factor 1 (SF1) identifies and attaches to BPS, while U2 small nuclear RNA auxiliary factor 1 and 2 (U2AF1 and U2AF2) attach to the conserved AG dinucleotide at the 3’ end of the intron and PPT, respectively. The U2AF heterodimer facilitates the substitution of SF1 with U2 small ribonucleoprotein (U2 snRNP) at the BPS after binding to the 3’ ss and PPT. The splicing factor 3B (SF3B), which is a protein subcomplex in the U2 snRNP, interacts with the nucleotide sequence that surrounds the adenosine in the BPS. U1 and U2 snRNP interactions bring the 5′ and 3′ splice sites into proximity. The secure attachment of U2 snRNP to pre-mRNA initiates the recruiting of tri-snRNP complexes comprising the U4, U5, and U6 complexes. Upon the presence of all five small nuclear ribonucleoproteins (snRNPs), the spliceosome experiences a conformational rearrangement, releasing U1 and U4. This sequence alteration initiates the stimulation of two transesterification reaction stages, leading to the splitting of the 5’ end and facilitating the joining of the 3’ end with the exon. Ultimately, introns are excised to produce mRNA, and snRNPs are liberated from the spliceosome complex and utilized in subsequent splicing cycles [7, 11–13](Fig. 1A).

Additionally, the interplay between cis-regulatory elements on the pre-mRNA and trans-acting splicing factor proteins modulates splice site recognition, influencing splicing accuracy (See Fig. 1B) [14]. Cis-regulatory elements include exonic splicing enhancers (ESEs), intronic splicing enhancers (ISEs), exonic splicing silencers (ESSs), and intronic splicing silencers (ISSs). Cis-regulatory elements encompass exonic and intronic splicing enhancers (ESEs and ISE) as well as exonic and intronic splicing silencers (ESS and ISS). These elements regulate exon inclusion in the final mRNA molecule. Serine/arginine-rich (SR) proteins and heterogeneous nuclear ribonucleoproteins (hnRNPs) are two prominent families of splicing factors that control alternative splicing by binding to regulatory regions in pre-mRNA.SR proteins facilitate the formation of spliceosomes and exon inclusion by recognizing ESEs [15].In contrast to SR proteins, hnRNPs can bind to ISSs and prevent the inclusion of exons [16]. Nevertheless, the diverse trans-acting factors that trigger the inclusion or exclusion of exons can have contrasting impacts based on the particular location where they bind [17–19]. The splice site selection process and the subsequent optimization of splicing can be influenced by the antagonistic or cooperative actions of these splicing factors.

AS is a process that generates multiple transcript variants from a single gene. This process expands protein diversity and phenotypic complexity [20]. Exons that are consistently present in the mRNA are referred to as constitutive exons, while exons that may occasionally be alternatively included in the mature mRNA are known as cassette exons. There exist seven fundamental types of AS events that can generate transcript variants (see Fig. 1C). Exon skipping is the predominant form of alternative splicing in higher eukaryotes, followed by an alternative 5’s or 3’s event. Retaining introns in mature mRNA is a more prevalent occurrence in plants and fungi. A Mutually exclusive exon refers to a pair of alternative exons where only one exon is included while the other exon is excluded. Ultimately, promoters or alternative polyadenylation sites on the first and last exons yield alternatively spliced transcripts [21, 22]. AS splicing regulates various cellular processes, and its dysregulation can drive tumor development or treatment resistance.

## Aberrant RNA splicing in cancer

Aberrant RNA splicing occurs in nearly all cancer types, driven by genomic changes and disruptions in splicing factors [5, 6]. Tumors exhibit up to 30% more alternative splicing events than normal tissues, generating cancer-specific splice isoforms [23]. These events can produce neoantigens that affect immune responses and hold potential for immunotherapy.

Mutations in splice sites, splicing factors, and spliceosome components (e.g., SF3B1, U2AF1, and SRSF2) disrupt splicing in cancer [4, 24–29]. Splice-site-creating mutations (SCM) in tumors are highly immunogenic, supporting their potential role in immunotherapy [30]. Mutations in U1 snRNA and synonymous mutations in key genes (e.g., TP53) also contribute to aberrant splicing, altering gene expression and function [31].

Aberrant expression of SFs further dysregulates AS in tumors. For example, SRSF1 is upregulated in lung, pancreatic, brain, and breast cancers, promoting isoform switching that drives tumor growth [32–35]. SRSF3, overexpressed in most solid tumors, enhances proliferation in breast, cervical, and nasopharyngeal cancers [36–39]. HnRNPA1, an hnRNP family member, regulate glycolysis by producing the PKM2 isoform, promoting tumor growth in multiple cancers. hnRNPA1 can also suppress tumor progression, inhibiting metastasis in gastric cancer [40–45]. The expression of these splicing factors plays a critical role in cancer progression, providing potential therapeutic targets.

**Fig. 2:**
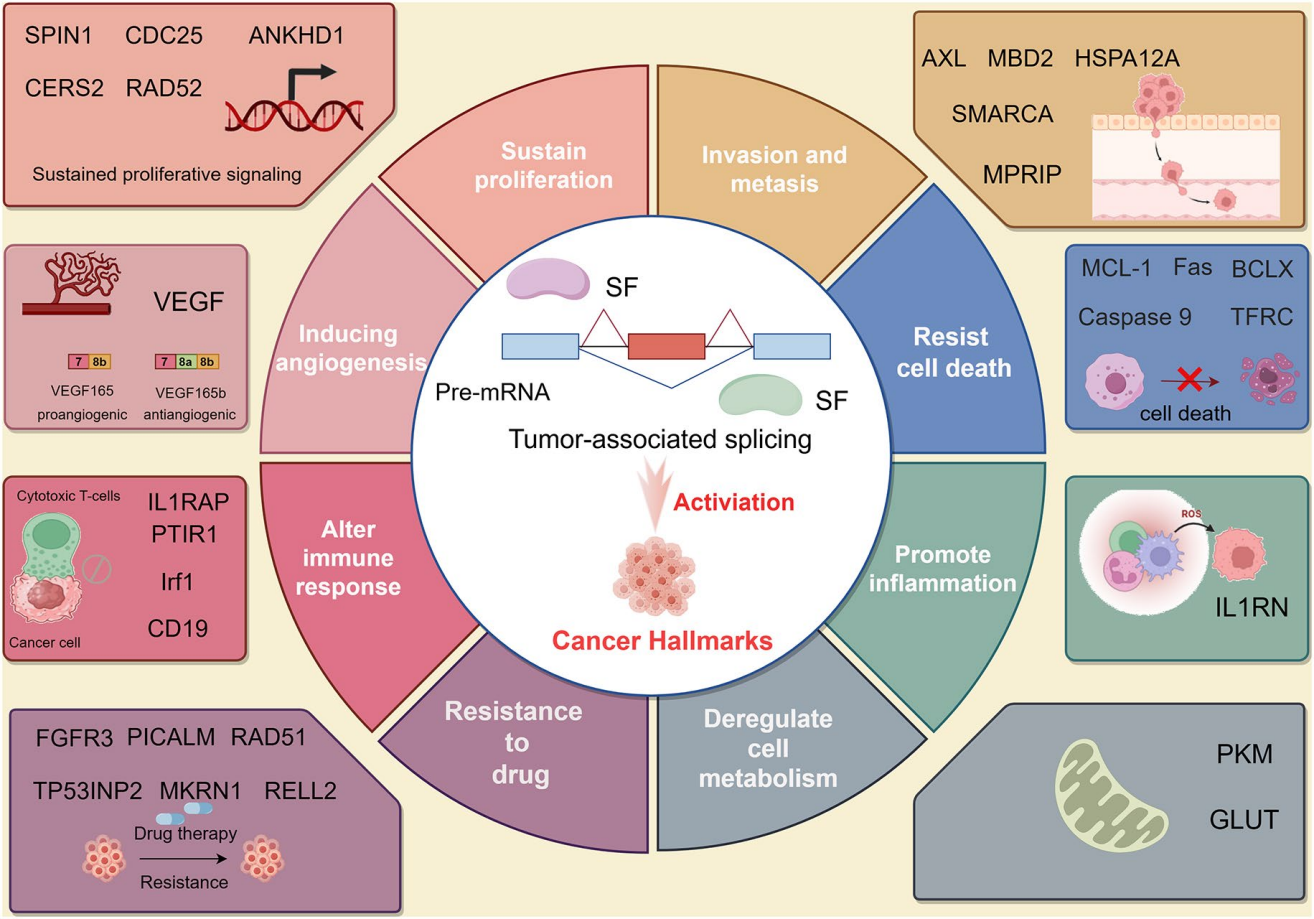
Tumor-associated splicing activates cancer hallmarks. Aberrant splicing can generate cancer-associated RNA isoforms, which activate cancer hallmarks. Including sustaining cell proliferation, tumor invasion and metastasis, inducing angiogenesis, resisting cell death, deregulating cell metabolism, promoting inflammation, altering the immune response, and resistance to drugs. This figure was drawn by Figdraw

Aberrant splicing generates cancer-specific RNA isoforms that drive hallmarks of cancer, including proliferation, metastasis, angiogenesis, immune evasion, and drug resistance (Fig. 2) [5]. Most of these cancer-associated AS events are regulated by different SFs (Table 1). For example, HNRNPK promotes SPIN1 exon 4 inclusion to regulate proliferation in oral cancer, while alternative isoforms of AXL and MBD2 enhance metastasis in liver and breast cancers [46–48]. Splicing of VEGF isoforms contributes to angiogenesis in ovarian and breast cancers [49, 50]. AS also affects cell death, with isoforms like MCL-1 S and BCL-xS promoting apoptosis [51, 52]. Additionally, splicing alters immune responses, as shown by CD19 exon 2 skipping, which impairs CAR-T therapy in leukemia [53]. Drug resistance is driven by isoforms like FGFR3-S and RAD51, which confer cancer therapy resistance [54, 55]. Understanding these splicing events offers opportunities for novel cancer treatments.

**Table 1:** SFs regulate AS of target genes to affect cancer hallmarks

Hallmarks	SFs	Target Pre-mRNA	Tumor type	Model	Refs
Sustain cell proliferation	HNRNPK	SPIN1	Oral squamous cell carcinoma	Cell line	[46]
	PUF60	CDC25	Lung cancer	Cell line	[63]
	SF3B4	RAD52	Ovarian cancer	Cell line	[64]
	-	CERS2	Breast cancer	Cell line	[65]
Invasion and metastasis	PTBP1	AXL	Liver cancer	Cell line, xenograft model	[47]
	SRSF2	MBD2	Breast Cancer	Metastasis, orthotopic models	[48]
	SRSF11	HSPA12A	Colorectal cancer	Xenograft model	[66]
	PTBP1	SMARCA	Colorectal cancer	Cell line, xenograft model	[67]
	RBFOX2	MPRIP	Pancreatic cancer	Xenograft model	[68]
Inducing angiogenesis	SRSF1	VEGF	Ovarian cancer	Xenograft models	[49]
	SRSF2	VEGFA (VEGF)	Breast cancer	Cell line	[50]
Resist cell death	SRSF1	MCL-1	Esophageal squamous cell carcinoma	Cell line	[51]
	SRSF6	Fas	Colon cancer	Cell line	[69]
	-	BCLX	Glioblastoma cells	Cell line	[52]
	SFPQ	Caspase 9	Ovarian cancer	Cell line	[70]
	RBFOX2	TFRC	Endometrial cancer	Cell line, Xenograft model	[71]
Alter immune response	SRSF10	IL1RAP	Cervical cancer	Cell lines, Xenograft model	[72]
	-	PTIRI	Colorectal cancer	SW480 cells	[73]
	-	IRF1	-	Th1 cells	[74]
	-	CD19	B-cell acute lymphoblastic leukaemia (B-ALL)	-	[53]
Promote inflammation	-	IL1RN	Intrahepatic Cholangiocarcinoma	Spontaneous mouse iCCA models	[75]
Deregulate cell metabolism	HnRNPA1	PKM	Lung adenocarcinoma	Xenograft models	[76]
	PTBP1	PKM, GLUT	Hepatocellular carcinoma	Cell line	[77]
Drug resistance	-	FGFR3	Prostate Cancer	Xenograft model	[54]
	SRSF6	PICALM	Gastric cancer	Tumor of PDX model	[78]
	YB1	RAD51	Colorectal cancer	Xenograft model	[55]
	HnRNPC	TP53INP2	Renal cell carcinoma	Xenograft model	[79]
	SF3A2	MKRN1	Triple-negative breast cancer	Cell lines, Xenograft model	[80]
	DHX38	RELL2	Pancreatic ductal adenocarcinoma	Cell lines	[81]

In addition to their role in RNA splicing, SFs have increasingly been recognized for their non-canonical functions that contribute to cancer development and progression. These factors influence various cellular processes, such as transcriptional regulation, chromatin remodeling, DNA repair, and RNA metabolism. For instance, some SFs interact with transcription factors and chromatin remodeling complexes to modulate gene expression [56, 57]. Similarly, some SFs are involved in DNA damage prevention and repair [58], for example, hnRNPK acts as a co-factor of p53, facilitating the repair of DNA double-strand breaks, thus promoting cancer cell survival and maintaining genomic integrity [59]. Additionally, SFs assist in microRNA processing, mRNA stabilization, and degradation [60–62]. These non-canonical functions offer new insights into cancer pathogenesis and present potential therapeutic targets for cancer treatment.

## Pharmacologic RNA splicing modulation

**Fig. 3:**
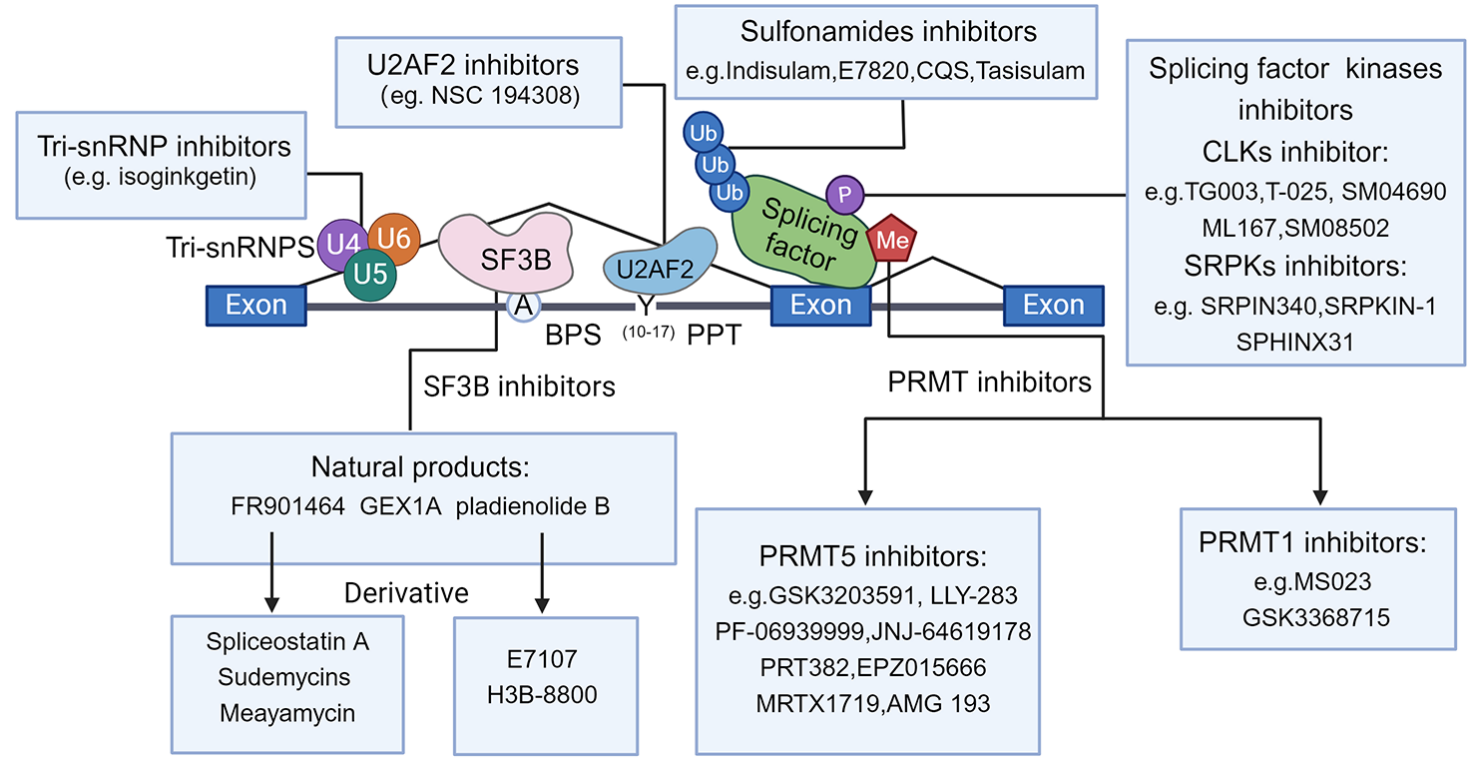
Small molecule drugs target RNA splicing strategies. Small-molecule drugs target RNA splicing. Inhibitors targeting the core spliceosome complex include inhibitors targeting Tri-snRNP, drugs directly targeting the core spliceosome complex SF3B, and inhibitors targeting U2AF2. Small-molecule drugs targeting the post-transcriptional modification of splicing factors include targeting ubiquitination, phosphorylation, and methylation. This figure was drawn by Biorender

Given the critical role of aberrant RNA splicing in cancer progression, targeting RNA splicing has emerged as a promising therapeutic for cancer. Small molecule drugs targeting RNA splicing are discussed, including those acting on the core spliceosome or enzymes modifying splicing factors (Fig. 3). The roles of these drugs in cancer therapy, from broad-spectrum RNA splicing regulation to specific isoform level changes, and their clinical prospects are also discussed.

### Directly target the core spliceosome

The spliceosome performs pre-mRNA splicing with exceptional accuracy. As above discussed, the association between cancer and the dysregulation of splicing is significant. Consequently, the identification of small molecules that specifically target spliceosome components presents a promising therapeutic prospect.

#### SF3B complex inhibitor

The SF3B complex is a critical spliceosome component for BPS and 3’ss selection, (see Fig. 1A), and restricting its function disrupts splicing at an early stage of spliceosome assembly. The SF3B complex includes the splicing factors SF3Bs family and PHD finger protein 5 A (PHF5A). A variety of natural products and derivative molecules targeting the SF3B complex have been identified and developed. For example, natural products such as FR901464 (Spliceostatin) [82], Pladienolides [83], GEX1A (Herboxidiene) [84], Thailanstatin A [85], and Jerantinine A [86] modulate RNA splicing and exhibit potent anticancer activity. Observing the anticancer properties of these natural products, synthetic analogues with improved chemical properties, such as E7107, H3B-8800, Spliceostatin A, Sudemycins, and Meayamycin, have been developed.

##### Natural products targeting SF3B complex

FR901464, originally extracted from the bacteria, exhibits potent in vitro anticancer activity against mouse and human tumor cell lines [82]. It binds to spliceosome components SF3B1 and PHF5A. Constantin Cretu and his colleagues used an FR901464 analogue to investigate how the U2 snRNP selects introns. They discovered that spliceostatin/Sudeomycin disrupted prespliceosome assembly and splicing fidelity by covalently binding PHF5A [87]. Pladienolide, a 12-membered macrolide from Streptomyces Mer-11107, interacts with the SF3B complex, hence impeding the process of RNA splicing [83]. The R1074H mutation in the SF3B1 reduces pladienolide’s binding affinity to the SF3B complex [ [88]. A new study has documented the crystal structure of human SF3B when it is bound to Pladienolide B (PB). PB acts as a wedge in a hinge, locking SF3B in the open conformation. This regulates SF3B’s transition to the closed conformation and allows it to stably accommodate the BPS/U2 duplex. This study clarifies the underlying structure that governs the splicing regulatory effects of PB and similar compounds. It uncovers key interactions between SF3B and shared pharmacophores, providing a basis for structure-based drug design [89]. GEX1A, a microbial product and natural splicing modulator, inhibits tumor growth. It targets the SAP155 protein (SF3B1), a critical protein in precursor mRNA splicing [84].

##### Derivatives of the natural products targeting SF3B complex

Based on the consensus pharmacophore derived from PB and FR901464, researchers developed a series of small molecule analogues. For example, E7107, a semisynthetic derivative of the natural product PB, inhibits the assembly of spliceosomes by blocking U2 snRNP binding to pre-mRNA [90]. H3B-8800, an orally available small molecule with preferential cytotoxicity, was identified by Michael Seiler et al. through an iterative medicinal chemistry endeavor based on a PB scaffold. These molecules potently compete with PB for binding to SF3B complexes [91]. FR901464 derivatives include Spliceostatin A, Sudemycins, and Meayamycin. Spliceostatin A hinders the process of splicing and enhances the accumulation of pre-mRNA by attaching to SF3B [92]. Sudemycins induce splicing alterations similar to those of Spliceostatin A [93]. Meayamycin is a highly effective antiproliferative drug that is more than 100 times stronger than FR901464 in inhibiting the growth of human breast cancer MCF-7 cells [94, 95]. Despite structural diversity and physicochemical properties of these compounds, these compounds target the SF3B complex in U2 snRNP and exhibit anti-tumor effects. This convergence highlights the potential of spliceosome-targeting drugs in anti-tumor therapy, encouraging further clinical exploration.

##### Anti-tumor mechanisms of SF3B complex inhibition

Altered splicing is emerging as a new cancer signature and a promising therapeutic target. Although splicing modulators elicit global effects, they specifically induce cancer cell death. This selectivity may arise that treatment with SF3B-targeting splicing modulators primarily affects the splicing events of a subset of genes involved in regulating the cell cycle and apoptosis [96–98]. Rocco Sciarrillo demonstrated that SF3B1 is emerging as a new potential prognostic factor for diffuse malignant peritoneal mesothelioma (DMPM). An investigation of differential splicing in cells treated with PB showed significant changes in the transcripts related to cell cycle, apoptosis, and other carcinogenic pathways [96]. Recently, Jacob P. Beard et al. synthesized Meayamycin D, which induces MCL-1 alternative splicing and exhibits anti-tumor specificity [99]. Emilia Alors-Perez et al. demonstrated that treatment with Pladienolide B in pancreatic ductal adenocarcinoma (PDAC) increased the expression of pro-apoptotic splice variants (BCL-XS, KRASa, Δ133TP53) and diminished tumor growth in zebrafish and mice [100]. In addition, SF3B-targeting splicing modulators also affect cancer characteristics such as proliferation, invasion, and stemness. For example, PB inhibits tumor proliferation, migration, and colony formation in hepatocellular carcinoma cells [101]. Another study revealed that the application of Pladienolide B in prostate cancer (PCa) can reduce the invasiveness of PCa cells and the viability of primary PCa cells [102]. Pladienolide B could also inhibit colorectal cancer cell proliferation and migration and TEAD2 splicing by targeting PH5FA [103]. GEX1A targets leukemic stem cells by inhibiting the production of FASTK mitochondrial isoforms, thereby repressing leukemia progression [104].

Overexpression or hyperactivation of MYC accelerates the synthesis of pre-mRNA, hence increasing the workload of the core spliceosome in its processing. Sudemycin D6 (SD6), an SF3B1 inhibitor, suppressed colony formation, induced MYC-dependent apoptosis, and inhibited tumor formation and metastasis in MYC-driven TNBC cells [105]. Another study linked splicing dysregulation to PCa progression and showed that E7107 efficiently suppressed the development of MYC-driven murine PCa as well as preclinical castration-resistant PCa models in vivo [106]. Collectively, these data suggest that pharmacologic suppression of the spliceosome is synthetically lethal with MYC.

Recent studies have demonstrated that pharmacologic modulation of splicing targeting SF3B may be preferentially lethal for cells bearing spliceosomal mutations in malignancies, compared to spliceosomal-wildtype cancers or normal cells. For example, H3B-8800 selectively kills epithelial and hematologic tumor cells with spliceosome mutations [91]. SF3B1 mutations can also mediate the sensitivity to H3B-8800 in chronic lymphocytic leukemia [107]. Another study demonstrated that hematopoietic cells with mutant U2AF1, including primary patient cells, are sensitive to sudemycin compounds [108]. Leukemias with SRSF2 mutations are more susceptible to the spliceosome inhibitor E7107 than wildtype counterparts [109]. Together, these data suggest that pharmacological inhibition of the spliceosome is synthetically lethal to malignancies carrying spliceosomal mutations.

E7107 and H3B-8800 underwent clinical trials but were subsequently terminated (refer to Table 2). E7107, a unique first-in-class molecule, was tested in a Phase I clinical trial where forty patients were enrolled. The inclusion criteria included patients with solid tumors refractory to standard therapies or those without available standard treatments. The most common side effects observed were gastrointestinal reactions. Following the cessation of the medication at a dosage of 4.0 mg/m^2^, one patient encountered temporary grade 4 visual impairment [110]. In another Phase I trial, the most prevalent drug-related side events were nausea, vomiting, and diarrhea. Notably, two patients suffered from vision loss, resulting in the termination of this trial [111]. The most frequent treatment-related side effects seen in a Phase I trial of the oral SF3B1 modulator H3B-8800 in myeloid neoplasms were diarrhea, nausea, lethargy, and vomiting. Nevertheless, there were no observed responses that fully or partially matched the criteria set by the International Working Group with or without core spliceosome mutations, however, nine patients experienced red blood cell transfusion independence [112]. This phenomenon may indicate that the dosage required to achieve cell killing exceeds the levels achieved in human subjects.

**Table 2:** Small molecule modulators of RNA splicing in cancer clinical trials

Classification	Drug	Target	Phase	Trial identifier	Disease	Year study started	Refs
SF3B inhibitors	E7107	SF3B	I	NCT00499499	Advanced solid tumors	2007-07	[111]
	H3B-8800	SF3B	I	NCT02841540	Myeloid neoplasms	2016-10	[112]
SAM- competitive PRMT5 inhibitors	JNJ-64,619,178	PRMT5	I	NCT03573310	Advanced malignant solid tumors or non-Hodgkin lymphomas (NHL)	2018-07	[136]
	PF-06939999	PRMT5	I	NCT03854227	Advanced Or metastatic solid tumors	2019-03	[133]
	PRT543	PRMT5	I	NCT03886831	Advanced solid tumors and hematologic malignancies	2019-02	[194]
MTA-cooperative PRMT5 inhibitors	MRTX1719	PRMT5	I/II	NCT05245500	Advanced solid tumors with homozygous MTAP Deletion	2022-06	[142]
	AMG 193	PRMT5	I/II	NCT05094336	Advanced MTAP^−^ solid tumors	2022-02	[147]
PRMT1 inhibitors	GSK3368715	PRMT1	I	NCT03666988	Advanced solid tumors	2018-10	[149]
CLKs inhibitors	SM08502	CLK1–3, DYRK1A, and DYRK1B	I	NCT03355066	Advanced solid tumors	2017-11	[166]
Sulfonamides inhibitors	E7070	RBM39	II	NCT00014625	metastatic melanoma	2001-02	[195]
	E7070	RBM39	II	NCT01692197	Relapsed or Refractory Acute Myeloid Leukemia and High-Risk Myelodysplastic Syndrome	2013-02	[185]
	E7820	RBM39	II	NCT05024994	Splicing factor mutant myeloid malignancies	2021-08	[181]
	Tasisulam	RBM39	III	NCT01006252	Metastatic melanoma	2009-12	[196]
	Tasisulam	RBM39	II	NCT00490451	Unresectable or metastatic soft tissue sarcoma	2007-08	[197]
	Tasisulam	RBM39	I	NCT01284335	Advanced solid tumors	2008-07	[187]

#### Targeting U2AF2 inhibitor

U2AF homology motifs (UHMs) and U2AF ligand motifs (ULMs) are critical domains for the interaction between U2AF2 and SFs [113]. During spliceosome assembly, U2AF2-SF1 and U2AF2-SF3B1 complexes are sequentially formed at the 3’ splice site via UHM/ULM interactions [114]. UHMCP1, a small molecule, affects RNA splicing and cell viability by targeting the U2AF2 UHM domain and disrupting the U2AF2/SF3B1 interaction [115]. In contrast, NSC 194,308 enhances RNA binding by the U2AF2 subunit, inhibiting splicing and stalling spliceosome assembly without blocking U2AF interactions. This inhibition occurs before tri-snRNP recruitment and catalytic activation, selectively killing leukemia cells harboring spliceosome mutations [116, 117].

#### Isoginkgetin inhibits recruitment of the tri-snRNP

Isoginkgetin, a natural biflavonoid isolated from Ginkgo biloba leaves, exhibits potent anticancer activity by targeting multiple SFs. It inhibits the spliceosome’s transition from the A complex to the B complex, thereby affecting pre-mRNA splicing globally [118]. By blocking this critical step in spliceosome assembly, Isoginkgetin induces widespread splicing defects, leading to the production of aberrant mRNAs and proteins. It also induces cell cycle arrest, particularly in S phase [119]. Additionally, Isoginkgetin disrupts glioblastoma cell growth, clonogenic potential, and migration via activation of apoptosis and autophagy [120]. Isoginkgetin also inhibits cancer cell invasion by downregulating matrix metalloproteinase-9 (MMP-9) via the PI3K/Akt/NF-κB pathway [121]. Furthermore, it synergizes with doxorubicin to inhibit hepatocellular carcinoma progression by activating autophagy through the AMPKα-ULK1 pathway [122]. Moreover, its water-soluble and non-toxic derivative, IP2, enhances the presentation of the nischarin-derived epitope on major histocompatibility complex (MHC) I molecules, thereby activating CD8^+^ T cells to recognize and eliminate tumor cells [123]. The multi-faceted actions of Isoginkgetin make it a promising candidate for further cancer therapy research.

### Target upstream regulator proteins

SFs and spliceosome proteins undergo extensive post-translational modifications. For instance, proteins in the spliceosome and SFs undergo significant arginine methylation. Numerous SFs, specifically SR proteins, undergo extensive phosphorylation. Phosphorylation events modify the function and localization of SFs, which are crucial for splicing activity. Therefore, modulating the methylation or phosphorylation of these spliceosome proteins and SFs may offer a viable strategy to curb oncogenic activity and open avenues for therapeutic intervention. The development of protein arginine methylation inhibitors and splicing protein kinase inhibitors targeting AS in cancer therapies, both in preclinical and clinical stages, is reviewed below.

#### Protein arginine methylation inhibitors

Directly targeting the spliceosome increases cancer cell vulnerability, highlighting the need to explore drugs with novel splicing modulation mechanisms. Blocking the post-transcriptional modification of splicing factors can indirectly disrupt RNA splicing by impairing spliceosome assembly and catalytic efficiency of splicing. For example, certain drugs can significantly perturb RNA splicing by inhibiting the asymmetric or symmetric arginine dimethylation mediated by type I or II protein arginine N-methyltransferases (PRMTs) [124, 125], especially PRMT1 and PRMT5 [126].

PRMT5 is essential for the assembly and normal splicing of spliceosomal UsnRNPs [127, 128]. Currently, numerous small molecule inhibitors targeting PRMT5 have been developed, exhibiting anti-tumor effects in a variety of solid and hematological tumors, with some advancing to Phase I or Phase II clinical trials (Table 2). Based on their mechanism of action, PRMT5 small molecule inhibitors are primarily categorized into substrate-competitive and S-adenosylmethionine (SAM) competitive inhibitors. With ongoing research and development, other types of PRMT5 inhibitors have been identified, including methylthioadenosine (MTA) cooperative PRMT5 inhibitors, PRMT degraders, and protein-protein interaction (PPI) inhibitors [129].

A recent report demonstrated that two orthogonal PRMT5 inhibitors, GSK3203591 and LLY-283, could inhibit the proliferation and self-renewal of glioblastoma (GBM) stem cells. This inhibition disrupted splicing across the transcriptome, particularly impacting the products of cell cycle genes [130].

PF-06939999, a selective SAM-competitive PRMT5 inhibitor, showed anti-proliferative activity in non-small cell lung cancer (NSCLC) cell models, primarily impacting the pathways responsible for regulating the cell cycle and alternative splicing. Additionally, PF-06939999 was found to sensitize NSCLC cell lines harboring splicing factor RBM10 mutations [131]. RBM10 mutations or deletions are the most common SF mutations in NSCLC, occurring at a rate of 8% [132]. These findings provide a crucial foundation for selecting patient populations for clinical trials involving PRMT5 inhibitors. Currently, PF-06939999 is in a Phase I clinical trial (NCT03854227), with 6 mg daily recommended as the dose for expansion, based on first-in-patient dose escalation data [133].

Another novel PRMT5 inhibitor, JNJ-64,619,178, exhibits sustained PRMT5 inhibition and significant antiproliferative action in a variety of cancer cell lines. In solid tumors, JNJ-64,619,178 has been reported to increase susceptibility to novel alternative splicing events [134]. These events generate novel open reading frames and subsequent expression of neoantigens, which may enhance the activation of immune cells and provide support for the utilization of JNJ-64,619,178 in conjunction with immunotherapies. A new study has identified that the core spliceosome protein SNRPD3 is vital for maintaining MYCN-driven AS events critical to neuroblastoma development. JNJ-64,619,178 efficiently decreases cell viability by inhibiting SNRPD3 methylation, which in turn impairs spliceosome activity in an SNRPD3- and MYCN-dependent manner [135]. Consequently, MYCN and SNRPD3 may serve as effective biomarkers for JNJ-64,619,178 in clinical treatments. JNI-64,619,178 is now being investigated in a Phase I trial (NCT03573310). The trial has shown first signs of toxicity and effectiveness against adenoid cystic carcinoma (ACC) and other solid tumors. Thrombocytopenia has been discovered as the only toxicity that limits the dosage [136]. However, no clinical benefit has been observed in myelodysplastic syndromes (MDS) patients [137].

These competitive SAM inhibitors, including LLY-283, JNJ-64,619,178, and PF-06939999, have shown limited specificity for PRMT5 and other methyltransferases, raising concerns about off-target effects on hematopoiesis. Consequently, PRT382, a selective PRMT5 inhibitor, was developed, offering improved tolerance and anti-tumor activity [138]. PRT-382 restores cell cycle regulation, induces cell death, and reactivates negative B-cell receptors regulators in Mantle cell lymphoma (MCL) [139]. Despite its anti-tumor activity, PRT-382 has shown drug resistance. Combining PRT-382 with the mTORC1 inhibitor Temsirolimus overcame resistance to PRMT5 inhibition and improved survival in resistant models, showing therapeutic synergy [140].

Methylthioadenosine phosphorylase (MTAP) is frequently subject to deletion in various types of human cancers, including approximately 50% of GBM [141], 40% of mesotheliomas [142], and 13% of NSCLC [143]. The absence of MTAP leads to the buildup of MTA, which competes with SAM for attaching to PRMT5 and functions as a specific inhibitor of PRMT5. Consequently, cancer cells with MTAP deletion are highly susceptible to PRMT5 inhibition [144]. EPZ015666 (GSK3235025), the first orally administered PRMT5 inhibitor, showed anti-proliferative effects in MCL. EPZ015666-treated MTAP^−^ cell lines showed lower IC_50_ values than in isogenic MTAP^+^ cell lines, suggesting greater sensitivity to PRMT5 inhibition [145]. Furthermore, PRT-382 is recommended for relapsed/refractory MCL, with MTAP/CDKN2A deletions and wild-type TP53 as biomarkers for favorable responses [139]. MRTX1719, a synthetic lethal inhibitor of the PRMT5 Complex, is used to treat tumors with MTAP deletion [146]. MRTX1719 is in a Phase I/II clinical trial for solid tumors with MTAP deletions. The study demonstrated anti-tumor activity in lung, pancreatic, and mesothelioma cancers, and provided partial early clinical data, highlighted by partial responses in six patients [142] A total of 39 patients with advanced MTAP-deleted solid tumors were enrolled in the phase I trial of the PRMT5 inhibitor AMG 193. Out of these patients, five exhibited a partial response following the administration of the initial medication [147]. This preclinical and early clinical data support a synthetic lethal strategy targeting PRMT5 in MTAP-deleted cancers.

It’s interesting to note that loss of PRMT1 makes cells more susceptible to PRMT5 inhibition [148]. The researchers discovered that the simultaneous use of PRMT1 inhibitor MS023 and PRMT5 inhibitor EPZ015666 resulted in a synergistic impact on the proliferation of lung cancer and pancreatic cancer [148]. Additionally, another study described a potent and reversible type I PRMT inhibitor, GSK3368715 (EPZ019997), with anti-tumor effects in human cancer models. Moreover, the combination of a PRMT5 inhibitor with GSK3368715 produced a synergistic tumor growth inhibition effect [125]. The drug GSK3368715 entered a phase I clinical trial, which was terminated early due to thromboembolic events [149].

#### Splicing factor kinases inhibitor

Two major kinase families, Cdc2-like kinases (CLKs) and SR-rich specific protein kinases (SRPKs), are primarily responsible for phosphorylating the arginine/serine dipeptide repeat domain of the serine and arginine-rich (SR) protein family [7, 11, 150]. SR proteins, modified by phosphorylation, regulate RNA splicing and participate in a variety of physiological functions [151].

**Fig. 4:**
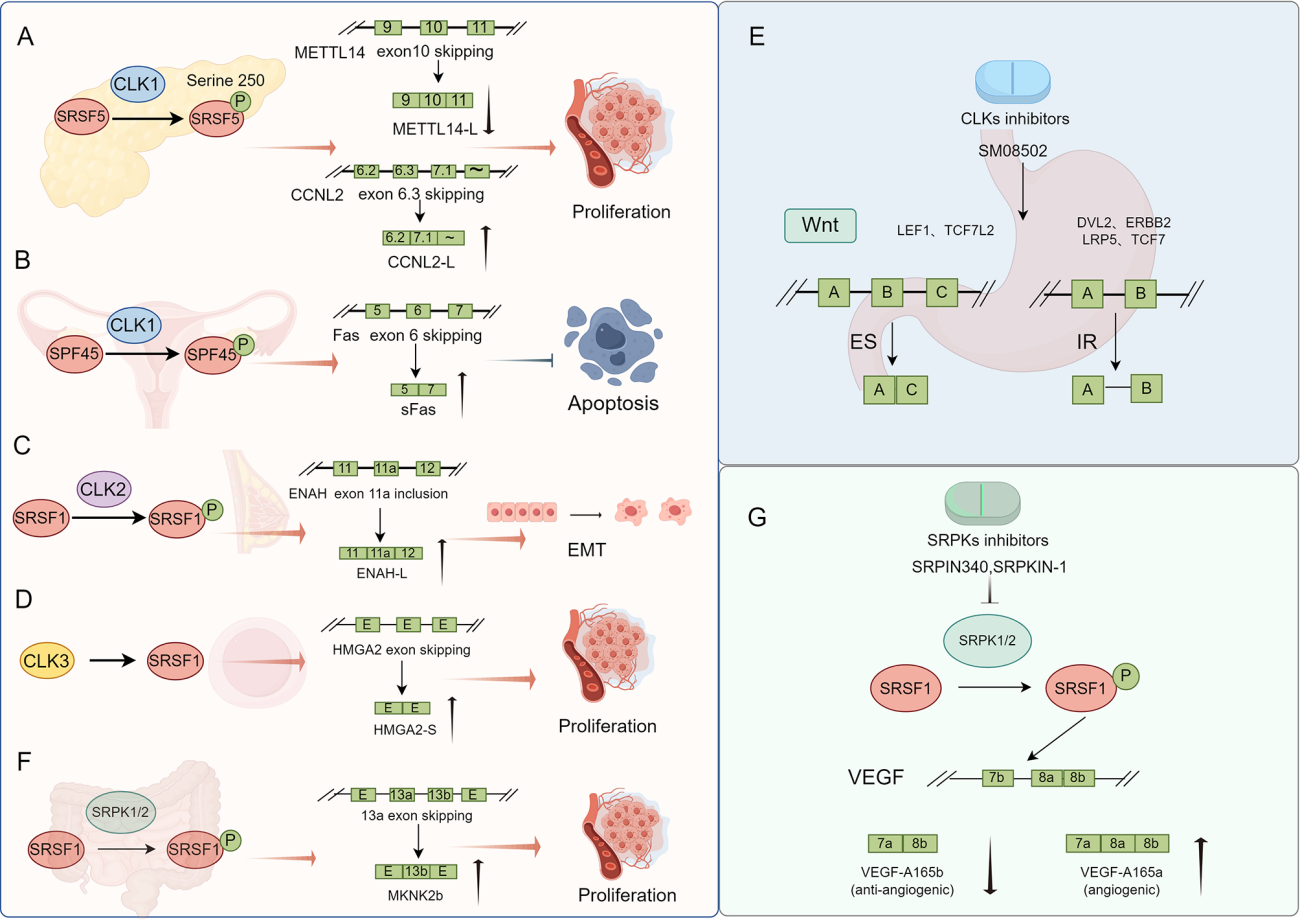
The regulation mechanisms of AS by two major kinase families and their inhibitors in tumors. (**A**) In pancreatic cancer, CLK1 modulates the phosphorylation of the splicing factor SRSF5, which affects the AS of METTL14 and CCNL2 and hence tumor cell proliferation. (**B**) In ovarian cancer, CLK1 regulates the phosphorylation of the splicing factor SPF45 and then affects the AS of Fas to inhibit tumor cell apoptosis. (**C**) CLK2 modulates the phosphorylation of the splicing factor SRSF1 in breast cancer, which influences ENAH’s AS and promotes EMT. (**D**) CLK3 affects the splicing factor SRSF1 and then affects the AS of HMGA2 to promote cell proliferation. (**E**) The CLKS inhibitor SM08502, which inhibits the Wnt pathway by inducing intron retention of DVL2, ERBB2, LPR5, and TCF7 and exon skipping of TCF7L2 and LEF1, has shown potent anti-tumor effects in gastrointestinal cancer. (**F**) In colon adenocarcinoma, SRPK1/2 promotes SRSF1 phosphorylation, promotes MKNK2 exon 13a skipping, and increases MKNK2b variants, which promotes tumor growth. (**G**) SRPK inhibitors SRPIN340 and SRPKIN-1 regulate SRSF1 phosphorylation and control VEGF AS. The antiangiogenic isoform VEGF-A165b contains exon 8b, and the proangiogenic isoform VEGF-A165a contains exons 8a and 8b. This figure was drawn by Figdraw

The CLK family comprises four isoforms (CLK1-4). CLK1 phosphorylates SRSF5 at serine 250, thereby inhibiting METTL14 exon 10 skipping and promoting Cyclin L2 exon 6.3 skipping. This aberrant splicing promotes PDAC cell proliferation (Fig. 4A) [152]. CLK1 can also phosphorylate the splicing factor SPF45, inducing the skipping of exon 6 of Fas precursor mRNA, generating sFas isoforms, inhibiting cell apoptosis, and promoting invasion and metastasis in ovarian cancer (Fig. 4B) [153]. TG003, a CLK1 inhibitor, reduces SRSF2 and pSRPK2 expression, suppressing cell proliferation and invasion in gastric cancer [154]. Transcriptome analysis reveals that TG003 therapy induces mis-splicing in cancer-related genes, including CD164, CENPE, and ESCO2 [155].CLK2 functions as an oncogenic kinase and splicing regulator [156]. CLK2 knockdown significantly reduced the phosphorylation level of the splicing factor SRSF1, promoted ENAH 11a inclusion, generated ENAH-L isoforms, and facilitated EMT in breast cancer (Fig. 4C). These results suggest that therapeutic targeting of CLK2 can modulate EMT splicing patterns and suppress breast tumor growth. Kenichi Iwai et al. designed a highly specific CLK2 inhibitor (T-025) that is stable, orally available, and exhibits anti-tumor activity in vivo. T-025 reduced the phosphorylation of SR proteins and consequently impaired RNA splicing (mainly inducing exon skipping), leading to reduced cancer cell growth, particularly in MYC-driven breast cancer [157]. SM04690, an novel intra-articular CLK2 inhibitor for the treatment of knee osteoarthritis, is in Phase II clinical trials [158]. A recent study reported that SM04690 promoted the alternative splicing of the Hippo pathway protein AMOTL2, producing exon-skipping products and activating YAP [159].

CLK3 is a dual-specificity kinase for serine/threonine and tyrosine substrates. Zhou et al. screened 1,280 compounds Collection for CLK3 inhibition and demonstrated that tacrine hydrochloride could be repurposed as a CLK3 inhibitor for cholangiocarcinoma (CCA) treatment [160]. A recent publication by Cesana et al. reported that CLK3 regulated HMGA2 splicing via SRSF1 in human hematopoietic stem cells (HSCs) [161]. CLK3 promotes HMGA2 exon skipping in an SRSF1-dependent manner, shifting the balance to HMGA2-S isoform production (Fig. 4D). Functional overexpression of HMGA2-S or CLK3, but not full-length HMGA2 (FL), restores proliferation and repopulation potential in adult CD34^+^ HSCs and progenitor cells [161]. HMGA2 is overexpressed in various types of cancer, such as lung cancer, gastric cancer, and breast cancer [162]. It also promotes cancer stem cell (CSC) properties in gastric and breast cancer [163, 164]. These discoveries highlight CLK3-SRSF1-HMGA2 splicing as a target for cancer therapy.

High CLK4 expression correlates with poor survival in TNBC patients, and CLK4 silencing inhibits TGF-β signaling-induced invasiveness and CSC properties. Importantly, the CLK4 inhibitor ML167 can effectively inhibit breast cancer cell invasion and proliferation [165].

SM08502, a novel CLK inhibitor, shows strong anti-tumor efficacy in a model of gastrointestinal cancer. Mechanistically, SM08502 inhibits the Wnt pathway by inducing intron retention in DVL2, ERBB2, LPR5, and TCF7 and exon skipping in TCF7L2 and LEF1 (Fig. 4E). SM08502 induces Wnt pathway gene level by significantly suppressing SRSF phosphorylation [166]. Orally administered SM08502 is in Phase I clinical trials, one study evaluating combination hormone or chemotherapy (NCT05084859) and the other assessing safety and pharmacokinetics as monotherapy (NCT03355066) (Table 2).

SRPKs, a family of kinases that regulate AS, are overexpressed in various cancers and promote the splicing of oncogenic isoforms of numerous genes. Some research has demonstrated that suppressing SRPKs can effectively decrease the proliferation of cancer cells, indicating their potential as therapeutic targets [167]. Overexpression of SRPK1/2 in colon adenocarcinoma (CAC) enhances SRSF1 phosphorylation, which subsequently leads to MKNK2 AS into MKNK2b, ultimately promoting tumor growth [168] (Fig. 4F). The SRPKs inhibitor SRPIN340, which regulates SRSF1 phosphorylation, controls VEGF alternative splicing and reduces the production of the pro-angiogenic isoform VEGF165 (Fig. 4G), reducing melanoma growth [169]. A recent study found that SRPIN340 enhanced immune response to metastatic melanoma in mice by upregulating MHC class I/II components. These findings provide insights into the functional roles of SRPKs in tumor biology [170]. Hatcher et al. described the SRPK1/2 inhibitor, SRPKIN-1, which blocked the phosphorylation of SR splicing factors protein. This led to a change in the isoform of the VEGF protein involved in blood vessel formation, transforming the pro-angiogenic VEGF-A165a to the anti-angiogenic VEGF-A165b isoform (Fig. 4G) [171]. Mussarat Wahid and colleagues demonstrated that SPHINX31, an SRPK1 inhibitor, could inhibit the phosphorylation of SRSF1. This inhibition resulted in AS and the production of the ∆Ex3PD1 variant of PD-1, which enhanced T-cell functionality in killing tumor cells. These findings suggest that small-molecule SRPK1 inhibitors could be a novel approach for drug-based immunotherapy [172].

### Target alternative splicing factors

Developing inhibitors that target specific splicing factors and RNA-binding proteins has been challenging. This challenge arises partly from the absence of catalytically active sites targeted by small molecule inhibitors. An interesting exception is the incidental discovery of sulfonamides with anticancer activity that inhibit cancer-associated splicing factors through previously unknown mechanisms. Multiple clinical investigations have demonstrated that aryl sulfonamides with selective anticancer activity are well tolerated. These sulfonamides act as molecular glues between RNA-binding motif protein 39 (RBM39) and the CUL4-DCAF15 E3 ligase, leading to the degradation of RBM39 [173–175]. Indisulam (E7070), Chloroquinoxaline Sulphonamide (CQS), Tasisulam, and E7820 have been confirmed to be effective molecular glues that specifically target RBM39 [173, 174, 176]. RBM39 works with U2AF65 and SF3B1 to coordinate spliceosome assembly and splice site recognition, acting as a coordinator for other regulatory SFs [174, 177].

These compounds have demonstrated excellent safety in clinical trials (Table 2) and have been proven to possess some anti-tumor effects. Most of these drugs are in phase II clinical trials; however, the overall response rates remain low. This may be due to limited understanding of both the mechanisms of action and the potential response biomarkers.

Recently, Wei-Ching Chen et al. discovered that DCAF15/RBM39 pathway regulates KRAS4A splicing and that inhibition of RBM39 by Indisulam reduces KRAS4A isoforms, suppressing CSCs [178]. Further screening is required to identify drugs that effectively target the CSC progenitors for improved treatments. In addition, it was demonstrated by Wang et al. that somatic SF mutations increased acute myeloid leukemia (AML) cells sensitivity to splicing inhibitors, suggesting these mutations as biomarkers for sensitivity to Indisulam and other sulfonamides [179]. Anke Nijhuis et al. demonstrated complete tumor regression without recurrence in a neuroblastoma model treated with Indisulam and showed that Indisulam’s dual targeting of metabolism and RNA splicing offers a promising treatment for high-risk neuroblastoma [180].

RBM39 is crucial for the viability of AML cells with SF mutations. A phase II clinical trial evaluated E7820 (100 mg daily) in myeloid malignancies carrying SF mutations [181]. The trial was terminated for futility as none of the first 12 patients enrolled achieved an objective response. Importantly, this study provides the first evidence that E7820 can induce RBM39 degradation and global changes in RNA splicing in patients. Preclinical studies have shown that pharmacological RNA interference can synergize with PARP inhibitors [182], BCL inhibitors [183], and immune checkpoint blockers [184] to enhance anti-tumor effects. Current findings support further exploration of combining E7820 with these agents. Additionally, in a phase II clinical trial, Indisulam combined with chemotherapy showed a 35% response rate in heavily pre-treated AML patients and was well-tolerated [185]. The anti-tumor activity of Indisulam is known to be dependent on the expression of DCAF15 and RBM39 [173, 186]. Therefore, DCAF15 and RBM39 may serve as biomarkers for assessing the efficacy of this treatment strategy. Moreover, a completed phase Ib study explored Tasisulam in combination with five standard chemotherapy agents—gemcitabine HCl, docetaxel, temozolomide, cisplatin, and erlotinib. This study provided preliminary anti-tumor activity for several combinations [187]. Although Tasisulam development was terminated, the study provided insights into the combined characteristics, toxicity, and function of the related potential mechanisms of synergy, offering future clinical development opportunities in specific tumor types.

### Target splicing variants

Some small molecule drugs targeting specific splicing variants have been developed to inhibit tumor progression. For example, Prodigiosin has shown efficacy in colorectal cancer by targeting the oncogenic isoform ΔNp73 [188], and BC-DXI-843 effectively induced tumor cell death by inhibiting AIMP2-DX2 in lung cancer [189]. JJ-450 inhibited androgen receptor (AR) and its variant ARv7 to suppress castration-resistant prostate cancer [190]. However, compounds targeting specific RNA transcripts for cancer therapy remain in the preclinical stage. Risdiplam, the first FDA-approved small-molecule drug for treating spinal muscular atrophy (SMA), functions by specifically targeting RNA transcription [191]. Mechanically, Risdiplam promotes the inclusion of SMN2 exon 7 by binding to exon 7 splice enhancer and the downstream intron of 5’SS in pre-mRNA, resulting in functional isoforms and therapeutic effects. Recent progress in splice-modifying drugs provides a basis for developing new therapies [192, 193]. These developments suggest that in the future, it may be feasible for small molecule drugs targeting cancer-related splicing variants to progress to the clinical stage for tumor treatment.

### Synergizes RNA splicing drugs for cancer therapy

#### RNA splicing drugs enhance anti-tumor immunity

Immune checkpoint blockade (ICB) therapy improves survival in several tumor types. While effective in more immunogenic tumors, ICB remains largely ineffective in tumors lacking immune cell infiltration, termed ‘cold tumors’. Combining ICBs with other treatments may improve the immunological conditions in the tumor microenvironment, thereby enhancing anti-tumor responses even in ICB-unresponsive tumors [184]. Pharmacological modulation of RNA splicing may enhance tumor sensitivity to ICB. A recent study demonstrated that, spliceosome-targeted therapies (STTs) induced tumor cell death, particularly in MYC-driven immune-cold TNBC. The small molecule spliceosome modulators H3B-8800 and the structurally distinct SD6 activate antiviral and adaptive immune signaling, inducing tumor cell death in immune-competent breast cancer models [198]. Another spliceosome modulator Pladienolide B, promotes cytotoxic immune cell infiltration and upregulates the expression of PD-L1, augmenting anti-tumor response in ovarian cancer [199] and providing preclinical evidence for the combination’s efficacy in ovarian cancer treatment.

PRMTs inhibition can also alter alternative RNA splicing [200]. In melanoma, upregulation of PRMT5 inhibits inflammation and antigen presentation. Combining PRMT5 inhibitor GSK3326595 with ICB therapy can limit melanoma growth and enhance immunotherapy efficacy in mice [201]. Additionally, in MYC-driven hepatocyte carcinoma (HCC), GSK3326595 promotes lymphocyte infiltration and enhances the expression of MHC II. Its combination with anti-PD-1 therapy can enhance the therapeutic efficacy of HCC [202]. The PRMT1 inhibitor GSK3368715 can increase T cell-mediated anti-tumor immune responses and sensitize immune-resistant tumors to PD-1 inhibition [203]. MS023, another PRMT1 inhibitor, acts synergistically with anti-PD-1 immunotherapy to enhance anti-tumor responses in TNBC mouse model [204].

A recent study conducted by Lu et al. proposed a new method to improve the effectiveness of ICB therapy [205]. This study demonstrated that pharmacological modulation of RNA splicing generated many immunogenic splice-derived neoantigens that enhanced the endogenous immune response post-ICB treatment. Two splicing regulating medicines, Indisulam and MS-023, were investigated at growth sub-inhibitory dosages. Simultaneous administration of Indisulam (or MS-023) and anti-PD-1 therapy dramatically suppressed tumor growth, outperforming either alone. These studies suggest that splicing modulators with diverse mechanisms can induce tumor neoantigens and enhance immunotherapy efficacy without genomic alterations, offering new directions for the clinical development of ICB therapy to improve responses in cold tumors.

#### RNA splicing drugs enhance response to BCL inhibitors

Therapy resistance presents a major challenge in cancer treatment. Combination therapy is widely used to circumvent acquired drug resistance in various cancer types. RNA splicing drugs targeting BCL family genes may offer novel strategies for BCL inhibitor-resistant patients. Eric Wang et al. demonstrated that the modulate RNA splicing drug SM09419, via inhibition of CLKs and DYRKs, enhances the response to BCL2 inhibition (Venetoclax) in leukemia [183]. Daniel Aird et al. identified that BCL2 genes vary in sensitivity to SF3b splicing modulators and that combining these modulators with BCLxL inhibitors induces synergistic cytotoxicity in cancer cells, thereby overcoming resistance [206]. Higher expression of MCL1 and BCL2A1 renders BCL2/BCLxL inhibitors ineffective. However, studies have found that E7107 can effectively downregulate MCL1 and BCL2A1, overcoming BCL2/BCLxL inhibitors resistance [206]. Additional research indicates that combining natural splice modulator GEX1A with the selective BCL-xL inhibitor blocks leukemic cell proliferation in an additive way in vitro [104]. H3B-8800 modulates MCL1 alternative splicing and displays synergistic effects with the BCL2 inhibitor Venetoclax in chronic lymphocytic leukemia (CLL) cells [107]. Consequently, strategies to reprogram apoptosis dependence via splicing modulators provide a rationale for clinical treatments, increasing patient susceptibility to BCL2 inhibitors.

#### RNA splicing drugs synergize with other inhibitors

In a recent study, investigators reported that combining PARP inhibitors with Indisulam represents a promising strategy for characterizing this combination therapy in terms of DNA damage repair and tumor growth [182]. It was observed that Indisulam inhibited Olaparib-induced DNA damage repair genes activation, and enhanced Olaparib’s efficacy. Another study found that Indisulam acted as an indirect CDK2 inhibitor and enhanced senescence in multiple cancers when combined with the CDK4/6 inhibitor Palbociclib [207]. CDK4/6 inhibitors induce retinoblastoma (RB) protein-mediated cell senescence, triggering the accumulation of immune cells, while splicing modulators can induce splicing errors to generate neoantigens [208]. Therefore, combining senescence induction with immunotherapy may be a potential therapeutic strategy in the future [209]. Suboptimal doses of CDK12/13 inhibitor THZ531 and the RNA splicing regulator Pladienolide B can synergistically suppress cell cycle progression and proliferation. These findings suggest that the combined application of kinase inhibitors and spliceosome inhibitors may offer a new exploitable anticancer approach with clinical relevance [210].

## RNA splicing modulation with ASOs

**Fig. 5:**
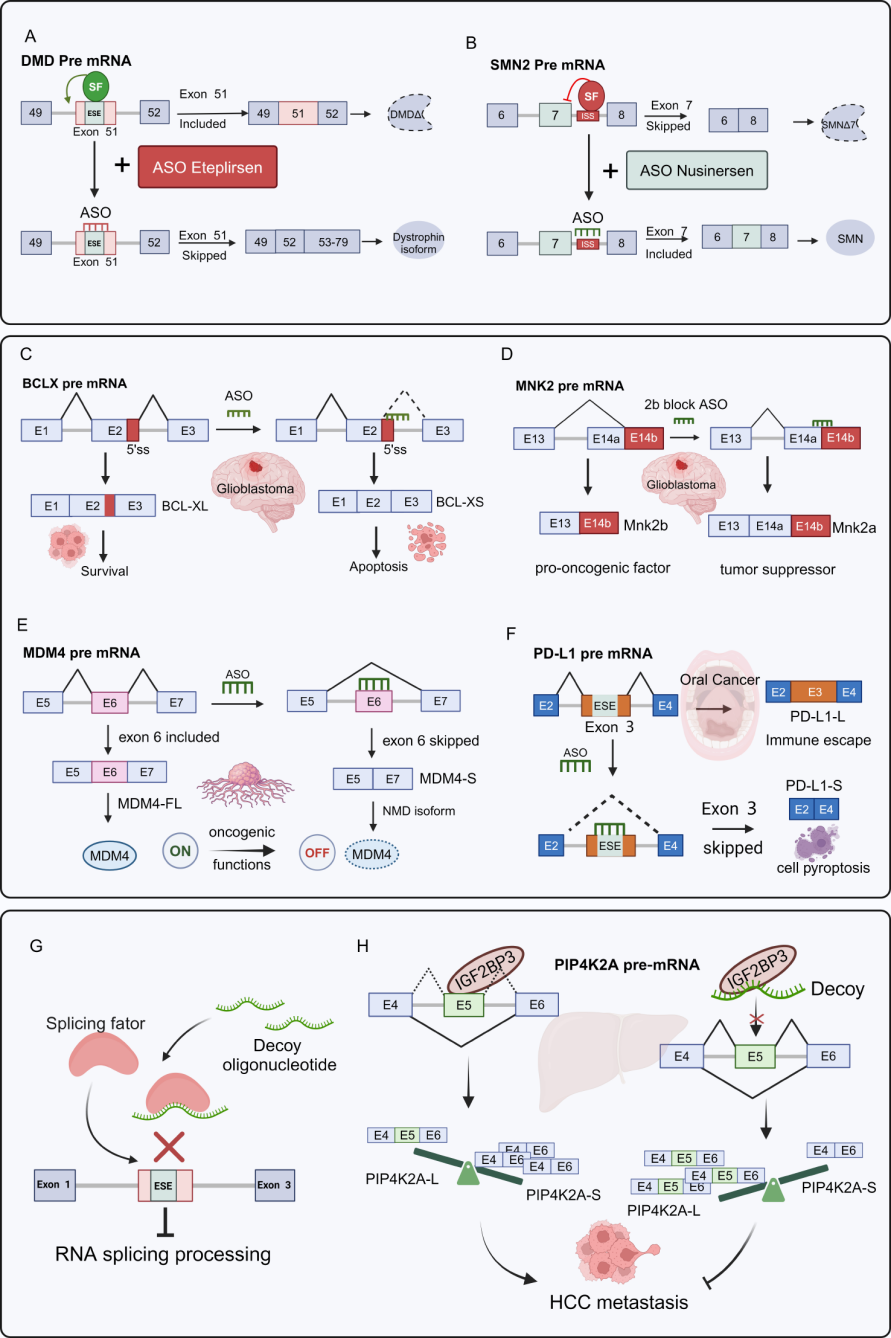
Splice-switching ASOs modulate AS as a therapeutic strategy. (**A**-**B**) Schematic of the mechanism of action of Eteplirsen and Nusinersen, ASOs drugs approved by the FDA for treatment. (**C**) In glioblastoma, ASOs targeted the 5’SS binding to exon 2 of BLC pre-mRNA, inducing a switch of AS from the BCL-XL isoform that promotes tumor growth to the BCL-XS isoform that promotes tumor cell apoptosis. (**D**) In glioblastoma, ASOs targeted binding to exon 14 of MNK2 pre-mRNA induced AS conversion from pro-oncogenic isoform MNK2b to tumor suppressor isoform MNK2a. (**E**) ASOs can target and bind to the exon 6 of MDM4 pre-mRNA and induce exon 6 skipping to produce the MDM4-S isoform to trigger NMD, which leads to a decrease in MDM4 protein levels and turns off the oncogenic switch. (**F**) In oral cancer, ASOs can target ESE that binds to exon 5 of PD-L1 pre-mRNA, enhance exon 3 skipping, generate PD-L1-S isoform, and promote tumor cell pyroptotic effect. (**G**) Schematic of the process of the decoy oligonucleotide inhibition RNA splicing. Decoy oligonucleotide to prevent a reaction between the RNA splicing factor and the ESE, inhibiting the RNA splicing process. (**H**) The decoy IGF2BP3 oligonucleotide interferes with the interaction between IGF2BP3 and PIP4K2A precursor mRNA, resulting in reduced expression of the exon 5-skipping PIP4K2A-S inhibition of HCC metastasis. This figure was drawn by Biorender

RNA therapy offers extraordinary specificity, with the ability to target virtually any sequence of pre-mRNA. Splice-switching ASOs are short, synthetic nucleic acids that bind to specific pre-mRNA regions, blocking splicing factor interactions. Recently, the splice-switching ASOs Eteplirsen [211] and Nusinersen [212] were approved by the FDA for the treatment of Duchenne muscular dystrophy (DMD) and SMA. Eteplirsen binds to exon 51 of DMD pre mRNA, leading to the skipping of exon 51 and restoration of the DMD open reading frame (Fig. 5A). Nusinersen binds to the intronic region of the exon 7 flanking sequence in SMN2 pre-mRNA, enhancing the inclusion of exon 7 and increasing functional SMN protein production to correct the disease (Fig. 5B). Currently, no splice-switching ASOs have been approved by the FDA for cancer therapy; the biggest challenge remains the delivery of ASOs to tumor tissues. Several preclinical studies have evaluated ASO-conjugated nanocarriers for cancer therapy [213]. For example, T7 peptides with high affinity for transferring receptors, are coupled to nanocarriers for specific tumor targeting in an A549 xenograft model [214].

While splice-switching ASO treatment for cancer is under evaluation, promising preclinical findings have emerged (see Table 3). Many studies have shown that splice-switching ASOs can correct cancer-associated alternative splicing, induce cell death [52, 215–222], and increase anti-tumor immune subtypes [223, 224], leading to tumor cell growth inhibition and regression in xenograft mouse models. For instance, ASOs induce the production of pro-apoptotic isoforms such as BCL-XS in glioblastoma [52] (Fig. 5C), BCL2L12-S [215] and BAX-L in ovarian cancer [216]. The gene encoding kinase Mnk2 (MKNK2) can be spliced to generate either a pro-oncogenic isoform Mnk2b or a tumor-suppressive isoform Mnk2a. ASOs that induce the Mnk2a isoform activate the p38 MAPK pathway, inhibiting oncogenic properties and reducing glioblastoma growth (Fig. 5D) [219]. Some ASOs induce exon skipping, triggering nonsense-mediated decay (NMD) that results in decreased protein levels and impairs tumor growth, such as in the cases of MDM4 [220] (Fig. 5E) and GLDC [221]). Resistance or transient responses to PD-1/PD-L1 immunotherapy in several patients are primarily attributed to tumor evasion from host immune surveillance. Lingyan Yan et al. determined that PD-L1 exon 3 is crucial for PD-L1 levels and surface translocation for immunosuppressive activity [223]. ASOs designed and optimized to significantly enhance exon 3 skipping, increased a PD-L1-S isoform, and decrease the levels of PD-L1-L isoform, along with an unexpected pro-pyroptotic effect in tumor cells (see Fig. 5F) [223].

**Table 3:** Splicing-modulating ASOs in cancer

Target gene	ASOs induced outcome	AS events	Tumor type	model	Refs
BCLX	Induce Bcl-xS activated apoptosis and autophagy	Bcl-xL; anti-apoptotic	Glioblastoma	Cell line	[52]
		Bcl-xS apoptotic			
BCL2L12	Reduced BCL2L12-L and increased BCL2L12-S and the cells subsequently undergo apoptosis.	BCL2L12-L (exon 3 inclusion) anti-apoptotic	Ovarian cancer	Cell line, xenograft model	[215]
		BCL2L12-S (exon 3 skipping) apoptosis			
BAX	Promote exon 2 inclusion and thus increase BAX expression, leading to inhibition of tumor growth	BAX-L (exon 2 inclusion) apoptosis	Ovarian cancer	Cell line, xenograft mouse model	[216]
		BAX-S PTC, (exon 2 skipping) anti-apoptotic			
PKM	Induce switch from the cancer-associated PKM2 to the PKM1 isoform, reversing the Warburg effect and inhibiting tumorigenesis	PKM2 (exon 9 inclusion) aerobic glycolysis	Hepatocellular Carcinoma	Cell line, xenograft mouse model	[217]
		PKM1(exon 10 inclusion) oxidative phosphorylation			
ERG	Induce exon 4 skipping, which resulted in reduction of ERG levels decreased cell proliferation, cell migration and increased apoptosis.	ERG (exon 4 skipping)	Prostate cancer	Cell line	[218]
MKNK2	Induce switch from the pro-oncogenic isoform Mnk2b to the tumor suppressive isoform Mnk2a, inhibited glioblastoma development	Mnk2a (inclusion exon 14a and exon 14b)	Glioblastoma	Cell line, xenograft mouse model	[219]
		Mnk2b (inclusion exon 14b)			
MDM4	ASO-mediated skipping of exon 6 decreased MDM4 abundance, inhibited melanoma growth	MDM4 (full-length) Suppress p53 tumor-suppressor function.	Melanoma	Cell line, xenograft (PDX) mouse model	[220]
		MDM4-S (exon 6 skipping) contains a premature termination codon and is targeted for nonsense-mediated decay (NMD)			
GLDC	Induce exon 7 skipping halt cell proliferation, and prevent colony formation	GLDC (exon7 skipping) disrupt the open reading frame (ORF) of GLDC transcript (predisposing it for NMD)	Non-small-cell lung carcinoma	Cell line, xenograft mouse model	[221]
HER4	Induce exon 26 Skipping generates CYT2 isoform inhibit cancer cell growth in vitro and in vivo	CYT2 isoform (HER4 pre-mRNA exon26 skipping)	Breast cancer	Cell line, xenograft mouse model	[222]
		CYT1 isoform (HER4 pre-mRNA exon26 inclusion)			
PD-L1	Block ESE of PD-L1, trigger exon 3 skipping enhanced immune cells’ suppression of cancer cell proliferation, inhibited cell growth and induced cell pyroptosis	PD-L1-L (full-length)	Oral cancer	Cell line	[223]
		PD-L1-S (exon 3 skipping)			
SLAMF6	Enhanced SLAMF6D^Δ17–65^ expression in human tumor-infiltrating lymphocytes and improved their capacity to inhibit human melanoma in mice	SLAMF6D^Δ17–65^ includes an alternative acceptor site, which consists of a 3’ alternative splicing of exon2, lacking amino acids 17–65 of the variable region	Melanoma	Cell line	[224]

Furthermore, Denichenko P et al. devised decoy Oligonucleotides that reduce splicing factor activity in the presence of up-regulation or hyperactivity of these factors (Fig. 5G). Decoy Oligonucleotides, such as the PTBP decoy, affect splicing and inhibit tumorigenic traits, targeting SRSF1 can suppress glioblastoma growth [225]. IGF2BP3 RNA decoy Oligonucleotides disrupt the interaction between IGF2BP3 and PIP4K2A pre-mRNA, reducing PIP4K2A‐S isoform with exon 5 skipping. This decrease has an inhibitory effect on the metastasis of HCC (Fig. 5H) [226].

## Conclusions and prospects

RNA splicing occurs cotranscriptionally in a complex dynamic process. Aberrant splicing contributes to cancer development, enabling cancer cells to survive, proliferate, and adapt to treatment. Advances in nucleic acid sequencing and computational biology have enhanced our understanding of the correlation between cancer and AS [227, 228]. The detection of AS can provide valuable biomarkers for cancer diagnosis, prognosis, and treatment. Thus, there is an urgent need to develop highly sensitive, specific, and cost-effective methods for the detection of alternative splicing isoforms.

To date, most studies have depended on short-read sequencing (SRS) technologies to characterize the AS repertoire in human tumors. Current SRS technology achieves high read depth but is unable to accurately detect complex alternative splicing [229]. Long-read sequencing (LRS) technologies can more accurately map full-length splice isoforms and quantify subtype-specific abundance but have low throughput, limiting its application to small genomes and transcriptomes [230]. With the increasing cost-effectiveness of LRS, it is anticipated that it will offer a more extensive perspective on the composition of alternatively spliced genes in both tumor and normal tissues. Obtaining the correct sequence of full-length splice isoforms is essential for identifying neoantigens. The potential use of neoantigens generated by such splicing abnormalities for personalized immunotherapy represents an emerging area in cancer therapy.

Tumors are genomically and transcriptomically heterogeneous, and AS exhibits similar complexity. The development of single-cell RNA sequencing technology (scRNA-seq) enables the identification of specific splice variants in heterogeneous tumor tissues [231]. Additionally, advances in spatial transcriptomics allow cell analysis within tissue context. The integration of spatial transcriptomics with scRNA-seq and LRS technologies has enhanced the characterization of functionally relevant heterogeneity [232]. The integration of these techniques offers a potent strategy to elucidate how AS influences tumor evolution and drug responses, as well as identifying tumor subgroups linked to drug resistance.

Despite advancements in measuring RNA splice isoforms, detecting and quantifying encoded protein isoforms remains challenging. Quantitative proteomics for detecting encoded protein AS isoforms [233] will further elucidate the functional roles of AS alterations in human malignancies and expedite the identification of novel therapeutic targets.

## Data Availability

No datasets were generated or analysed during the current study.

## Abbreviations

pre: mRNA-Precursor messenger RNA
AS: Alternative splicing
ASO: Antisense oligonucleotide
snRNAs: Small nuclear RNAs
5′ss: 5′ splice donor site
3′ss: 3′ splice acceptor site
BPS: Branch point site
PPT: Polypyrimidine tract
SF1: Splicing factor 1
U2AF1: U2 small nuclear RNA auxiliary factor 1
U2AF2: U2 small nuclear RNA auxiliary factor 2
U2 snRNP: U2 small ribonucleoprotein
SF3B: Splicing factor 3B
ESEs: Exonic splicing enhancers
ISEs: Intronic splicing enhancers
ESSs: Exonic splicing silencers
ISSs: Intronic splicing silencers
SR: Serine/arginine-rich
hnRNPs: Heterogeneous nuclear ribonucleoproteins
SCM: Splice-site-creating mutations
TP53: p53 tumor suppressor gene
SFs: Splicing factors
EMT: Epithelial-mesenchymal transition
HNSCC: Head and neck squamous cell carcinoma
NPC: Nasopharyngeal carcinoma
VEGF: Vascular endothelial growth factor
iCCA: Intrahepatic cholangiocarcinoma
HR: Homologous recombination
MKRN1: Makor in ring finger protein 1
TNBC: Triple-negative breast cancer
PHF5A: PHD finger protein 5 A
PB: Pladienolide B
DMPM: Diffuse malignant peritoneal mesothelioma
PDAC: Pancreatic ductal adenocarcinoma
PCa: Prostate cancer
RRMs: RNA recognition motifs
PRMTs: Protein arginine N-methyltransferases
SAM: S-adenosylmethionine
MTA: Methylthioadenosine
PPI: Protein-protein interaction
GBM: Glioblastoma
NSCLC: Non-small cell lung cancer
ACC: Adenoid cystic carcinoma
MDS: Myelodysplastic syndromes
MCL: Mantle cell lymphoma
MTAP: Methylthioadenosine phosphorylase
CLKs: Cdc2-like kinases
SRPKs: SR-rich specific protein kinases
CCA: Cholangiocarcinoma
HSCs: Hematopoietic stem cells; CSC-cancer stem cell
CAC: colon adenocarcinoma
RBM39: RNA-binding motif protein 39
AML: Acute myeloid leukemia
AR: Androgen receptor
SMA: Spinal muscular atrophy
ICB: Immune checkpoint blockade
STTs: Spliceosome-targeted therapies
HCC: Hepatocellular Carcinoma
RB: Retinoblastoma
DMD: Duchenne muscular dystrophy
MKNK2: Kinase Mnk2
NMD: Nonsense-mediated decay
